# Supplementary material for: Priority Attachment: a Comprehensive Mechanism for Generating Networks
Source: Sci Rep. 2019 Mar 4;9:3383. doi: 10.1038/s41598-019-40015-9 (PMC6399255; doi:10.1038/s41598-019-40015-9)
Supplement: Supplementary file 1 — Supplementary File [file 41598_2019_40015_MOESM1_ESM.pdf]

Priority Attachment: a Comprehensive Mechanism for Generating Networks

Mikołaj Morzy[1*], Tomasz Kajdanowicz[2], Przemysław Kazienko[2], Grzegorz Miebs[1], and Arkadiusz Rusin[1]

[1]Institute of Computing Science, Poznań University of Technology, 60-965 Poznań, Poland
[2]ENGINE - The European Centre for Data Science, Faculty of Computer Science and Management, Wrocław University of Science and Technology, 50-370 Wrocław, Poland
[*]Mikolaj.Morzy@put.poznan.pl

Empirical networks

In this section we present the descriptions of empirical networks used in the experimental evaluation of the Priority Rank model. All networks are available through The Colorado Index of Complex Networks[1] and The Network Repository.[2] Directed networks are marked with (D) while undirected ones with (U).

- **American bisons** (D): Dominance relations within a group of American bisons in the National Bison Range in Moiese Montana observed in 1972[3] are described in the first real world network. Each vertex corresponds to a single animal and edges to dominance of the source vertex animal over the target vertex animal. Vertices in the original network lack of any attributes, so we do not have any insight into which animal features are responsible for the dominance.

- **Bighorn sheep** (D): The network represents dominance interactions among a group of female bighorn sheep from the National Bison Range in Montana observed over the 27 month period in 1984.[4] Vertexes represent animals, whereas edges are derived from the dominating behavior of the source animal towards the target vertex individual. In addition, each animal is described by a single numerical attribute representing its age.

- **C.elegans** (D): The network contains connections between neurons and synapses of the C.elegans nematode.[5] It is significantly larger than the previous empirical networks.

- **Mouse visual cortex** (D): The network depicts a very small group of neurons and their interactions in the mouse primary visual cortex recorded using electron microscopy.[6] This small network is very difficult to recreate, most probably due to the fact that its generative process may be complex. Unfortunately, the source network does not contain any additional attributes and we have to rely only on topological features.

- **Enzyme 108** (U): This small network represents chemical interactions between a small set of enzymes. The main challenge when trying to recreate this network are the very large diameter and very long average shortest path.

- **Cage5** (D): The network studied in this experiment has been originally published by van Heukelum *et al.* as a part of the study on DNA electrophoresis.[7]

- **Political books** (D): The network captures the co-purchasing patterns of books on U.S. politics published close to the 2004 U.S. presidential election and sold on Amazon.[8] Vertices represent book titles and edges represent frequent co-purchasing of these books.

- **Primary school** (U): The network reflects contacts between students and teachers at a primary school in Lyon, France, collected in October 2009. Each vertex represents either a student or a teacher, and an edge – any type of contact between the two persons.[9] The main challenge that this network poses is the density of social interactions and the very short average shortest path length.

- **Vickers 7th graders** (D): This is a small social network of friendship among seventh grade students in Victoria, Australia.[10] Each vertex represents a student and each edge corresponds to the nomination of the target vertex student in one of three categories: getting on with in the class, being a best friend, being preferred to work with.

- **Freeman researchers** (D): The network describes letters exchanged by researchers studying social networks in 1978.[11] An interesting feature of this network are communication patterns in the pre-internet era. As expected, the network is characterized by very high reciprocity, small diameter and small average shortest path length.

- **9/11 terrorists** (U): This is the network of individuals and their social associations, centered around the terrorists who hijacked the planes on the September 11th, 2001.[12] All data has been gathered and extracted after the terrorist attack from publicly available data.
- **Zachary karate club** (U): This is the classic social network of friendship among members of the university karate club; collected and compiled by Zachary.[13]
- **Illinois high school** (D): The network represents the structure of friendship relationships among students in a small high school in Illinois as recorded in 1957 and 1958.[14] It is a very typical network of interpersonal ties that appear in small, tightly connected communities. The most distinguishing feature of this network is its very large diameter and relatively long average shortest path. Theoretically, these networks tend to follow the small world network model of Watts and Strogatz.
- **Marseille high school** (D): The main difference between this network and the network of Illinois high school students is the fact that the contacts and friendships between students in a high school in Marseille, France, have been measured through sensors and questionnaires.[15] Each student has been described by two categorical attributes: the class which a given student belongs to and their sex.
- **CAG** (D): The main focus of this experiment is to check if the Priority Rank model is capable of capturing the generative process of highly atypical network induced by integer matrices used in computations of characteristic polynomials.[16]
- **Power network** (U): This dataset representing the structure of power stations connections is challenging due to its very low density, large diameter and lack of degree centralization.
- **Football** (D): The network represents the exchange of professional soccer players among countries participating in the 1998 World Championships in Paris. Each vertex denotes a country and existence of an edge means that the source vertex country is exporting players to the target vertex country. The network has been compiled by Lothar Krempel and presented during 2001 Dagstuhl seminar "Link Analysis and Visualization".
- **St.Marks ecosystem** (D): The network depicts the carbon flow between species in St.Marks National Wildfire Refuge, Florida.[17] Vertices represent species and edges denote the flow of carbon (mostly due to predation) between species.

Distance function search

Below we present an outline of the approach to brute force distance function search. We would like to remind the reader, that the brute force approach is rudimentary and will be supplemented in the near future by the machine learning approach of automatic distance learning. With respect to the results presented in this paper, however, the following protocol has been followed. The only hyperparameter of the method is the length of the ranking (the number of possible target vertices for a given vertex). For each distance function presented in Table 2, we have tried ranking lengths of $[10\%, 20\%, 30\%, 40\%, 50\%]$ of the number of vertices n, computing the similarity of resulting network with the original network. For the best performing ranking length we have then tried several values of the ranking length around that value. We present the results obtained for all distance functions for a single network N_1. As expected, many distance functions miss the mark in a big way, and only some are able to re-create the original network to a sufficient degree. We want to stress again that this result is temporary as the brute force approach will be supplemented in the near future by a machine learning model responsible for automatic determination of the most suitable distance function.

References

1. Clauset, A., Tucker, E. & Sainz, M. The colorado index of complex networks. `http://icon.colorado.edu` (2016).
2. Rossi, R. A. & Ahmed, N. K. The network data repository with interactive graph analytics and visualization. In *Proceedings of the Twenty-Ninth AAAI Conference on Artificial Intelligence* (2015). URL `http://networkrepository.com`.
3. Lott, D. F. Dominance relations and breeding rate in mature male american bison. *Ethology* **49**, 418–432 (1979).
4. Hass, C. C. Social status in female bighorn sheep (ovis canadensis): expression, development and reproductive correlates. *Journal of Zoology* **225**, 509–523 (1991).
5. White, J. G., Southgate, E., Thomson, J. N. & Brenner, S. The structure of the nervous system of the nematode caenorhabditis elegans: the mind of a worm. *Phil. Trans. R. Soc. Lond* **314**, 1–340 (1986).
6. Bock, D. D. *et al.* Network anatomy and in vivo physiology of visual cortical neurons. *Nature* **471**, 177 (2011).

p_D	p_B	p_C	$\|V\|$	$\|E\|$	d	ρ	L	$\rho^2_{C_D}$	network
			100	300	14.00	0.03	4.32	0.02	original
**0.91**	0.00	0.00	100	300	11.00	**0.03**	5.05	0.03	random
0.00	0.00	0.00	100	300	4.00	**0.03**	2.07	0.33	degree
0.00	0.00	0.00	100	300	4.00	**0.03**	1.78	0.28	betweenness
0.00	0.00	0.00	100	300	3.00	**0.03**	1.64	0.36	closeness IN
**0.99**	0.00	0.00	100	300	21.00	**0.03**	7.98	**0.02**	closeness OUT
0.00	0.00	0.00	100	300	5.00	**0.03**	1.94	0.32	closeness ALL
0.00	0.00	0.00	100	300	4.00	**0.03**	1.70	0.34	pagerank
**1.00**	0.00	0.00	100	300	18.00	**0.03**	6.55	**0.02**	dissimilarity - simple
**0.91**	0.00	0.00	100	300	16.00	**0.03**	5.62	0.03	dissimilarity - advanced
0.00	0.00	0.00	100	300	**13.00**	**0.03**	**4.90**	0.06	cosine
0.00	0.00	0.00	100	300	4.00	**0.03**	2.12	0.27	regression
0.00	0.00	0.00	100	300	5.00	**0.03**	2.11	0.30	naive bayes
**1.00**	**0.11**	**0.05**	100	300	**14.00**	**0.03**	**4.60**	**0.02**	euclidean

Table 1. Re-creation of an exemplary network. Instances where the null hypothesis (samples drawn from single distribution) cannot be rejected are marked with bold font, as well as scalar network descriptors such as diameter d or density ρ that are regenerated within $\pm 10\%$ margin of the original value

7. van Heukelum, A., Barkema, G. & Bisseling, R. Dna electrophoresis studied with the cage model. *J. Comput. Phys.* **180**, 313–326 (2002). URL http://dx.doi.org/10.1006/jcph.2002.7095.
8. Krebs, V. Political books and polarised readers. *A New Political Pattern Emerges* (2006).
9. Stehlé, J. *et al.* High-resolution measurements of face-to-face contact patterns in a primary school. *PloS one* **6**, e23176 (2011).
10. Vickers, M. & Chan, S. Representing classroom social structure. *Victoria Institute of Secondary Education, Melbourne* (1981).
11. Freeman, S. C. & Freeman, L. C. *The networkers network: A study of the impact of a new communications medium on sociometric structure* (School of Social Sciences University of Calif., 1979).
12. Krebs, V. E. Mapping networks of terrorist cells. *Connections* **24**, 43–52 (2002).
13. Zachary, W. W. An information flow model for conflict and fission in small groups. *Journal of anthropological research* **33**, 452–473 (1977).
14. Coleman, J. S. *et al.* Introduction to mathematical sociology. *Introduction to mathematical sociology.* (1964).
15. Fournet, J. & Barrat, A. Contact patterns among high school students. *PloS one* **9**, e107878 (2014).
16. Lo, S., Monagan, M. & Wittkopf, A. Strongly connected graph components and computing characteristic polynomials of integer matrices in maple. *preprint* (2006).
17. Baird, D., Luczkovich, J. & Christian, R. R. Assessment of spatial and temporal variability in ecosystem attributes of the st marks national wildlife refuge, apalachee bay, florida. *Estuarine, Coastal and Shelf Science* **47**, 329–349 (1998).
